# HKU1 immune imprinting is associated with post-COVID symptoms after SARS-CoV-2 infection

**DOI:** 10.1016/j.isci.2026.115175

**Published:** 2026-03-02

**Authors:** Abdelilah Majdoubi, Christina Michalski, Allison W. Watts, Xiaoqing Dang, S. Amirhossein Golzan, Bahaa Abu-Raya, Sirui Li, Jacob Shew, Frederic Reicherz, Louise C. Mâsse, Pascal M. Lavoie

**Affiliations:** 1 British Columbia Children’s Hospital Research Institute, Vancouver, British Columbia V5Z 4H4, Canada; 2 Department of Pediatrics, Faculty of Medicine, University of British Columbia, Vancouver, British Columbia V6T 1Z4, Canada; 3 Department of Pediatrics, Faculty of Medicine, Dalhousie University, Halifax, Nova Scotia B3H 4R2, Canada; 4 Canadian Center for Vaccinology, Dalhousie University, Halifax, Nova Scotia B3K 6R8, Canada; 5 Department of Pediatrics, Children’s Hospital Datteln, University of Witten/Herdecke, 58455 Witten, Germany; 6 School of Population and Public Health, University of British Columbia, Vancouver, British Columbia V6T 1Z3, Canada

**Keywords:** Health sciences, Medicine, Immunology, Virology

## Abstract

The long-term health burden of SARS-CoV-2 infections remains poorly understood. In a cohort from Vancouver, Canada, we identified immune imprinting to endemic β-human coronaviruses (HCoVs), reflected by affinity-matured IgG responses that cross-reacted with the SARS-CoV-2 spike with low affinity, along with an early expansion of memory B cells recognizing HKU1 and the conserved S2 domain following the first dose of ancestral-strain vaccination. Vaccination also enhanced antibody-dependent cellular phagocytosis (ADCP), primarily directed against the HKU1 spike and SARS-CoV-2 S2 domains. In another cohort from the same region, higher HKU1 spike IgG levels and increased antibody-dependent complement deposition (ADCD) were associated with a greater likelihood of post-COVID symptoms, even though these individuals had experienced fewer SARS-CoV-2 infections at the one-year follow-up. Together, these findings suggest that β-HCoV-associated immune imprinting may simultaneously reduce infection risk and promote pathological Fc-mediated inflammation, potentially contributing to post-COVID conditions following infection with contemporary SARS-CoV-2 variants in individuals vaccinated against earlier strains.

## Highlights

• SARS-CoV-2 vaccination recalls low-avidity human β-coronaviruses antibodies
• The recalled B cells preferentially target conserved epitopes on the S2 domain
• HKU1 cross-reactive antibodies show enhanced Fc-mediated effector function
• These antibodies increased post-COVID symptoms in two independent cohorts

## Graphical abstract

**Figure:**
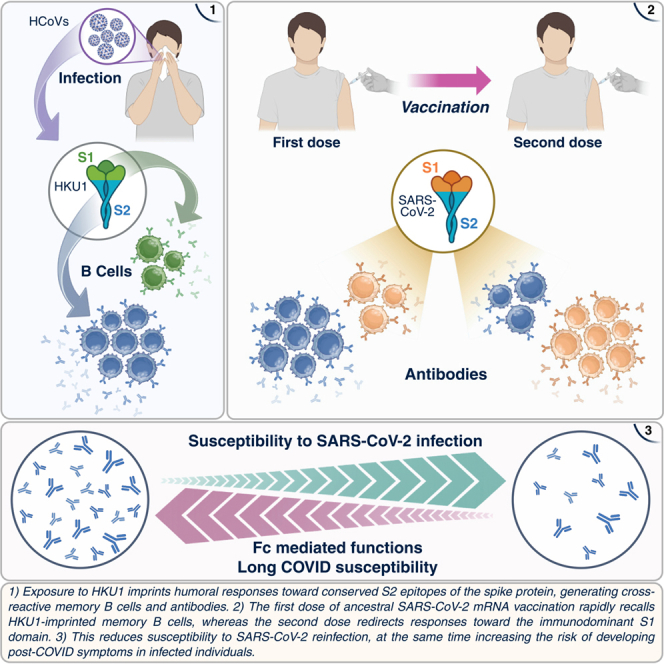

## Introduction

SARS-CoV-2 has become endemic, and continues to circulate globally as new variants emerge.^1^ Although vaccines provide strong protection against severe disease, hospitalization, and death, they do not fully prevent infection, partly due to viral immune evasion.^2^^,^^3^^,^^4^^,^^5^^,^^6^ Moreover, while vaccination reduces the risk of developing post-COVID conditions,^7^ increasing evidence suggests that repeated reinfections may carry a small but significant cumulative risk of developing chronic post-COVID symptoms in individuals vaccinated against ancestral SARS-CoV-2 strains.^8^^,^^9^^,^^10^ Recent population-based studies estimate that 5–15% of adults infected with SARS-CoV-2 experience post-COVID symptoms, such as fatigue, cognitive impairment, dyspnea, and cardiovascular complications.^11^^,^^12^^,^^13^ Globally, post-COVID syndromes affect approximately 400 million individuals.^14^ In Canada, 16.7% of adults with confirmed or suspected SARS-CoV-2 infection experienced post-COVID symptoms (https://www.statcan.gc.ca/en/covid19). These findings highlight the need to better understand the immune correlates of chronic post-infectious syndromes.

Immune imprinting refers to the tendency of the immune system to rely on pre-existing memory B cells generated in response to prior antigens, thereby limiting the development of B cell responses against novel antigens.^15^^,^^16^^,^^17^^,^^18^^,^^19^^,^^20^^,^^21^^,^^22^^,^^23^^,^^24^^,^^25^ While this mechanism can sometimes enhance immune protection, it can also contribute to viral immune evasion and lead to either beneficial or pathological outcomes.^26^ In the context of SARS-CoV-2, immune imprinting frequently targets the receptor-binding domain (RBD) of the spike protein, a key site of antibody neutralization.^15^^,^^16^^,^^17^^,^^18^ This has enabled emerging variants with RBD mutations to evade neutralizing antibodies and potentially cause reinfections, even in vaccinated individuals.^2^^,^^3^^,^^4^^,^^5^^,^^6^ Beyond the RBD, the S2 domain of the spike protein contains epitopes that are highly conserved across endemic human coronaviruses (HCoVs), particularly the beta-coronaviruses HKU1 and OC43, and to a lesser extent, alpha-coronaviruses (229E and NL63).^27^^,^^28^^,^^29^^,^^30^ Lifelong exposure to commonly circulating HCoVs induces durable antibody responses.^31^^,^^32^ These observations raise important questions about how cross-reactive immunity targeting the conserved S2 domain may influence immune imprinting toward new SARS-CoV-2 variants. Specifically, they suggest a potential mechanism through which prior exposure to HCoVs may shape pathological immune imprinting responses to SARS-CoV-2.

Several studies have shown that pre-existing immunity to HCoVs can influence the outcome of infection and vaccination.^33^^,^^34^^,^^35^ SARS-CoV-2 infection has been associated with increased antibody levels against the spike protein of HKU1 and OC43,^36^^,^^37^^,^^38^^,^^39^^,^^40^^,^^41^^,^^42^^,^^43^ supporting the concept of immune imprinting between SARS-CoV-2 and β-HCoVs.^44^ However, few studies have examined the clinical relevance of these findings in the context of post-COVID-19 symptoms. Given that imprinting toward β-HCoVs drives antibody responses that target conserved epitopes with Fc-mediated effector functions,^45^^,^^46^ we hypothesize that such antibodies contribute to pathological Fc receptor-dependent immune activities.

Here, we investigated the immune and clinical consequences of HCoV-related imprinting, using data and biological samples from the two adult cohorts in the Vancouver region, Canada. During the primary SARS-CoV-2 vaccination series, the first vaccine dose induced an early (≤14 days) increase in low-avidity antibodies targeting the HKU1 spike in the first cohort. Memory B cells recognizing the conserved S2 domain were detectable at baseline and expanded earlier than those targeting the S1 domain, coinciding with an early rise in HKU1 spike-specific B cells. Following the second vaccine dose, B cell responses shifted toward the immunodominant S1 domain, mirroring previously reported immune imprinting toward SARS-CoV-2 variants.^9^ Notably, antibodies targeting both the SARS-CoV-2 S2 domain and the HKU1 spike exhibited stronger Fc receptor-mediated function than those directed against the SARS-CoV-2 S1 domain. In the second cohort, HKU1 spike antibodies were associated with a reduced risk of SARS-CoV-2 infection from emerging variants. Paradoxically, however, higher HKU1 IgG levels were also associated with increased post-COVID symptoms among those who became infected. Altogether, these results suggest that while HKU1 spike-specific immune imprinting may confer protection against infection, it may also contribute to the development of post-COVID symptoms through enhanced inflammatory Fc-mediated mechanisms, such as complement deposition.

## Results

### Primary SARS-CoV-2 vaccine series induces a recall of low-avidity antibodies against HKU1 spike

**Figure 1:**
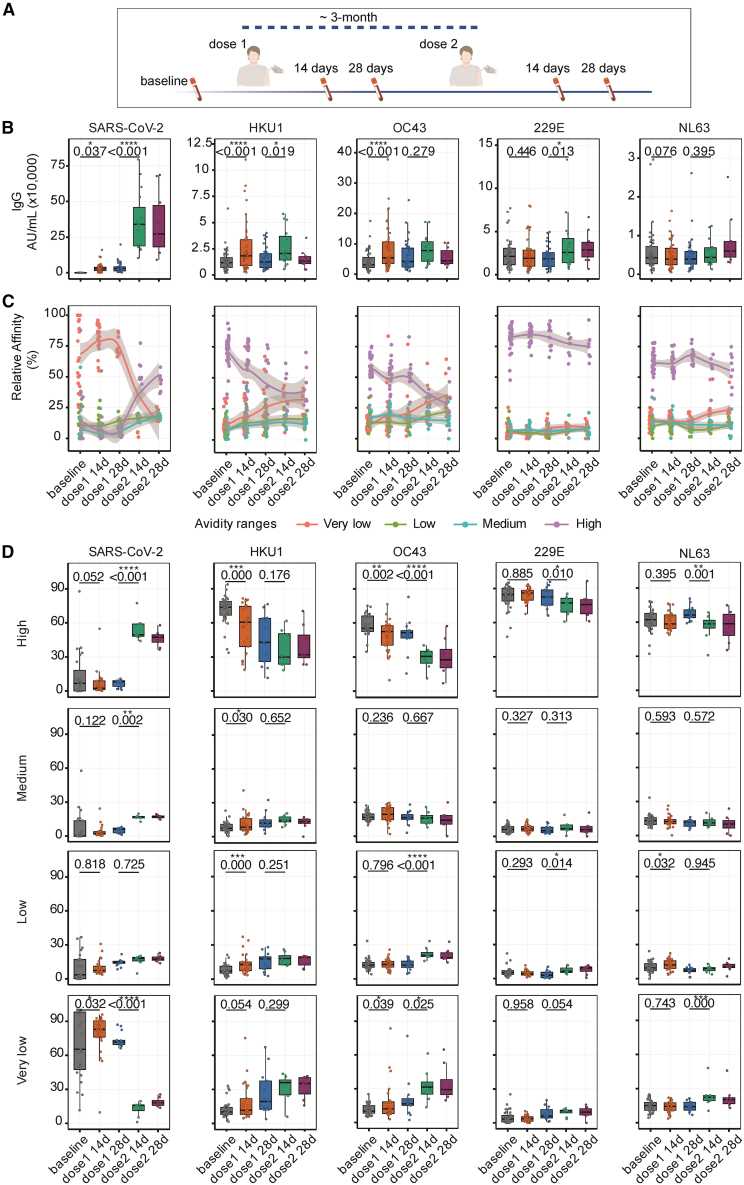
SARS-CoV-2 vaccination boosts pre-existing antibody reactivity to human coronaviruses (HCoVs) (A) Timeline of vaccine administration and peripheral blood sampling in the first (healthcare workers) cohort. (B) IgG responses to the spike protein of SARS-CoV-2 and HCoVs (OC43, HKU1, NL63, and 229E) expressed as arbitrary units per milliliter (AU/mL), shown as box (median, with lower/higher quartiles)-and-whiskers (upper whisker: Q3+1.5xIQR, lower whisker: Q1-1.5xIQR) plots, with all data points. (C) Relative IgG avidity profiles against SARS-CoV-2 and HCoV spike, across time points, with LOESS-smoothed trend lines, showing all data points and 95% confidence intervals (gray shade). (D) Corresponding avidity ranges for SARS-CoV-2 and HCoV spike antigen, shown as box-and-whisker plots (similarly defined as in [B]), with all data points. Paired comparisons were conducted between baseline and 14 days after the first vaccine dose, and between 28 days after the first dose and 14 days after the second dose, using a mixed-effects model (∗*p* < 0.05, ∗∗*p* < 0.01, and ∗∗∗*p* < 0.001). Sample sizes: baseline (*n* = 48), 14 days post-first dose (*n* = 46), 28 days post-first dose (*n* = 35), 14 days post-second dose (*n* = 15), and 28 days post-second dose (*n* = 11).

In a previous study, we reported frequent antibody cross-reactivity between the spike proteins of SARS-CoV-2 and HKU1 in a cohort of 276 immunologically naive, unvaccinated healthcare workers from Vancouver, BC, Canada.^47^ To investigate the functional consequences of this cross-reactivity for immune imprinting, we examined recalled HCoV responses after a primary SARS-CoV-2 vaccination series in 48 adults from the original cohort (randomly selected from 96 uninfected individuals invited to participate in this follow-up study), followed longitudinally through their first ancestral-strain mRNA vaccine series. Individuals in this first cohort received their two doses of SARS-CoV-2 mRNA vaccine at a standard 3-month interval, in accordance with Canadian public health recommendations at the time. Demographic characteristics of the participants, including age and sex distribution, are shown in Table S1. The interval between the two vaccine doses was 98 days (median; IQR: 95–104), allowing us to track the evolution of antibody and B cell responses over a three-month period (Figure 1A and Table S1).

The first vaccine dose induced a modest but significant increase in SARS-CoV-2 spike-specific IgG, followed by a more robust boost after the second dose (Figure 1B). Notably, a significant, rapid rise in HKU1 and OC43 spike IgG antibodies was detected 14 days after the first dose, with no significant change in NL63 or 229E IgG antibodies (Figure 1B). In contrast to the SARS-CoV-2 response, HKU1 and 229E-specific antibody levels marginally increased further after the second dose. These findings are consistent with a recall response to HCoVs following infection or vaccination described by others.^35^^,^^37^^,^^41^^,^^48^

We next assessed the impact of SARS-CoV-2 vaccination on the avidity profile of IgG antibodies targeting spike from HCoVs and SARS-CoV-2, using an ammonium thiocyanate-based chaotrope antibody avidity assay described earlier.^49^^,^^50^ Data showed that most antibodies exhibited very low avidity against the SARS-CoV-2 spike at baseline and after the first dose. In contrast, high-avidity SARS-CoV-2 spike antibodies were detected after the second dose (Figure 1C), suggestive of antibody affinity maturation against SARS-CoV-2 spike.^51^^,^^52^ Conversely, antibodies displayed high avidity against HCoV spike proteins at baseline and this avidity decreased after the second dose of SARS-CoV-2 vaccination (Figure 1C). Interestingly, this shift in antibody avidity was mainly observed for HKU1 and OC43 (Figure 1C). After the second dose, relatively little change was detected in the avidity profile of HCoV-specific antibodies (Figures 1C and 1D). Altogether, these findings indicate that vaccination with the SARS-CoV-2 ancestral strain boosted low-avidity, cross-reactive antibodies against β-HCoVs, potentially recognizing shared spike epitopes.

### Evidence of HKU1 immune imprinting primarily targeting the SARS-CoV-2 spike S2 domain

Next, we used flow cytometry to define the antigenic target of this imprinting response. Given that the S2 domain is more conserved between SARS-CoV-2 and HCoV spike proteins, whereas the S1 domain, including the RBD, is substantially different structurally,^25^^,^^30^^,^^53^^,^^54^^,^^55^ we examined whether recalled responses to the first vaccine dose preferentially induced antibodies to the SARS-CoV-2 S1 or S2 domains. We assessed S1- and S2-specific B cell responses before and after vaccination, using surface markers to identify plasmablasts, total memory B cells (MBCs), and IgG^+^ MBCs specific to the SARS-CoV-2 S1 and S2 domains, as well as the HKU1 spike (see Figure S1 for representative gating).

**Figure 2:**
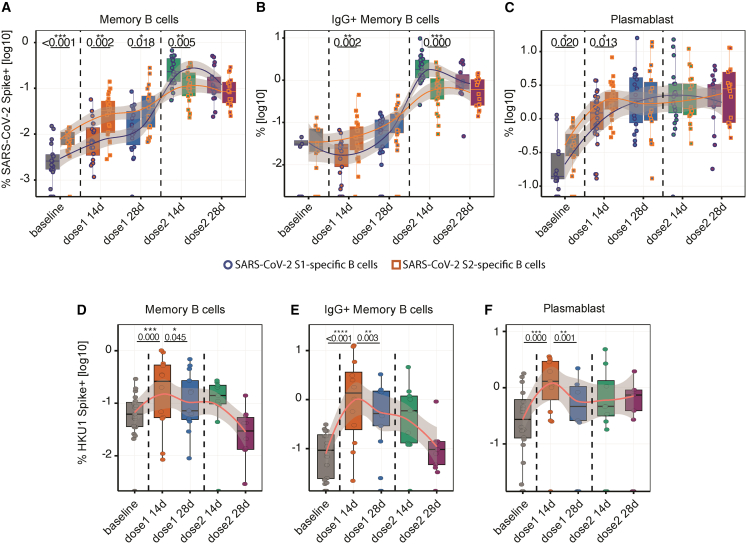
HKU1 immune imprinting predominantly targets the SARS-CoV-2 S2 domain Percentages of antigen-specific memory B cells, IgG^+^ B cells, or plasmablasts calculated as the number of antigen-specific cells within each subset divided by total B cells, IgG^+^ B cells, or plasmablasts, respectively, across time points (flow cytometry). Log10-transformed data are shown as box (median, with lower/higher quartiles)-and-whiskers (upper whisker: Q3+1.5xIQR, lower whisker: Q1-1.5xIQR) plots, with all data points, and LOESS-smoothed trend lines with 95% confidence intervals (gray shade). (A–C) Percentage of S1 and S2-specific cells within memory and IgG+ memory B cells, and plasmablasts. Comparisons were performed between S1 and S2 groups using a Wilcoxon signed-rank test. (D–F) Percentages of HKU1-specific B cells within total and IgG+ memory B cells, and plasmablasts. Comparisons assessed between sequential vaccine time point pairs, using mixed-effects models (only significant results are shown; ∗*p* < 0.05, ∗∗*p* < 0.01, and ∗∗∗*p* < 0.001). Sample sizes: baseline (*n* = 20); 14 days (*n* = 30) and 28 days post-first dose (*n* = 19); and 14 days (*n* = 22) and 28 days post-second dose (*n* = 18).

At baseline, individuals exhibited higher proportions of SARS-CoV-2 S2-reactive MBCs, IgG^+^ MBCs, and plasmablasts compared to their S1-reactive counterparts. The first vaccine dose induced expansion of both S1 and S2-reactive MBCs and plasmablasts. However, S2-reactive B cells remained more abundant than their S1 counterparts, with statistical significance observed up to 14 days after the first SARS-CoV-2 vaccine dose. The second vaccine dose preferentially induced S1 and S2-reactive MBCs and IgG^+^ MBCs but did not affect the proportions of S1 and S2-reactive plasmablasts. Notably, within 14 days after the second vaccine dose, S1-reactive IgG^+^ MBCs dominated the responses, compared to S2-reactive IgG^+^ MBCs (Figures 2A–2D). These findings suggest that the first vaccine dose primarily induced a recall of S2-reactive MBCs, whereas the second vaccine dose shifted the immune response toward S1-reactive MBCs.

The rapid recall of β-HCoV spike antibody responses following the first SARS-CoV-2 vaccine dose prompted questions about their origin. We previously showed that cross-reactive SARS-CoV-2 spike antibodies correlated with HKU1, but not OC43, in this cohort.^47^ Because HKU1-specific antibodies were also preferentially boosted by vaccination, we used flow cytometry to identify HKU1-specific B cells. Accordingly, HKU1 spike-specific IgG^+^ MBCs and plasmablasts expanded at 14 days after the first vaccine dose (Figures 2E–2G). No further increase in HKU1 spike-specific B cells was observed after the second vaccine dose, again supporting a heterologous recall response. Moreover, this early expansion of S2-reactive B cells that temporally coincided with an early increase in low-avidity HKU1 spike IgGs (Figure 1D) supported immune imprinting targeting the conserved S2 domains.

### Immune imprinting elicited against HKU1 enhanced antibody-dependent cellular phagocytosis

**Figure 3:**
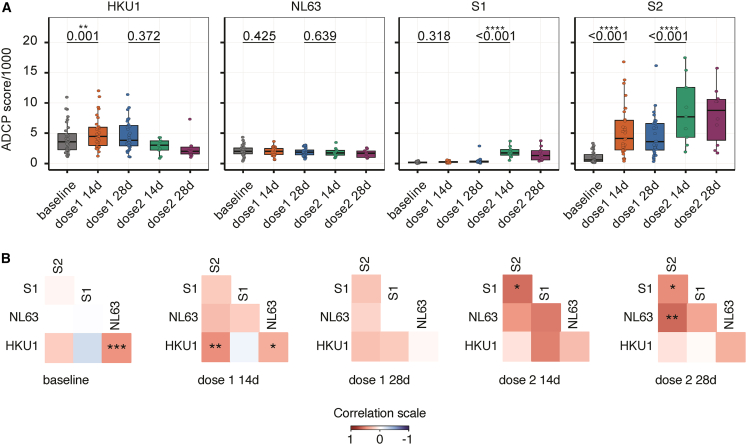
Recalled HKU1 spike antibody responses display enhanced ADCP function (A) Antibody-dependent cellular phagocytosis (ADCP) responses against HCoV spike and SARS-CoV-2 S1- and S2-domains across vaccine doses and time points. ADCP scores were compared between baseline and 14 days post-first dose, and between 28 days post-first dose and 14 days post-second dose, using a mixed-effects model. Data are shown as box (median, with lower/higher quartiles)-and-whiskers (upper whisker: Q3+1.5xIQR, lower whisker: Q1-1.5xIQR) plots, with all data points. (B) Spearman correlations between HKU1 and NL63 spike, and SARS-CoV-2 S1- and S2-domain-specific ADCP responses, adjusted for multiple comparisons with the Benjamini-Hochberg method. Spearman correlations are shown as a heatmap for each time point. (only significant results are shown; ∗*p* < 0.05, ∗∗*p* < 0.01, and ∗∗∗*p* < 0.001). Sample sizes: baseline (*n* = 48), 14 days post-first dose (*n* = 46), 28 days post-first dose (*n* = 35), 14 days post-second dose (*n* = 15), and 28 days post-second dose (*n* = 11).

Next, we examined antibody-dependent cellular phagocytosis (ADCP) to assess the functional impact of antibodies targeting the HKU1 and SARS-CoV-2 S1 and S2 spike domains. As shown in Figure 3A, ADCP activity mediated by HKU1 spike antibodies increased significantly 14 days after the first SARS-CoV-2 vaccine dose. In contrast, no change in antibody-mediated ADCP was observed for the distantly related NL63 spike, which served as an α-HCoV control not expected to be affected by vaccination. These findings suggest a specific increase in Fc receptor-mediated effector function directed against the HKU1 spike in this cohort. Notably, the increase in ADCP was more pronounced for SARS-CoV-2 S2-specific antibodies than for S1-specific antibodies (Figure 3A). Furthermore, HKU1- and SARS-CoV-2 S2-specific antibody-mediated ADCP responses were strongly correlated after the first vaccine dose, whereas no correlation was observed between SARS-CoV-2 S2 and NL63 spike-specific ADCP at the same time points (Figure 3B). Together, these findings support an HKU1-specific immune imprinting response to SARS-CoV-2 mRNA vaccination in this cohort.

### HKU1 antibodies protect against SARS-CoV-2 infection but increase the risk of developing post-COVID symptoms after infection in fully vaccinated individuals

To evaluate the clinical relevance of the HKU1-related immune imprinting, we analyzed a second, independent cohort of fully vaccinated adults prospectively enrolled from the same Vancouver metropolitan area. SARS-CoV-2 infection and post-COVID symptom rates over one year following the emergence of the first Omicron variant were examined in relation to HKU1 antibody reactivity levels. This second cohort showed an infection seroprevalence of 26.5% at the beginning of the study period.^10^^,^^56^ To exclude prior SARS-CoV-2 infection as a confounder, we restricted analyses to infection-naïve individuals at baseline, confirmed by nucleocapsid serology. At the end of the one-year follow-up period, participants prospectively reported post-COVID symptoms by questionnaire.^57^

**Figure 4:**
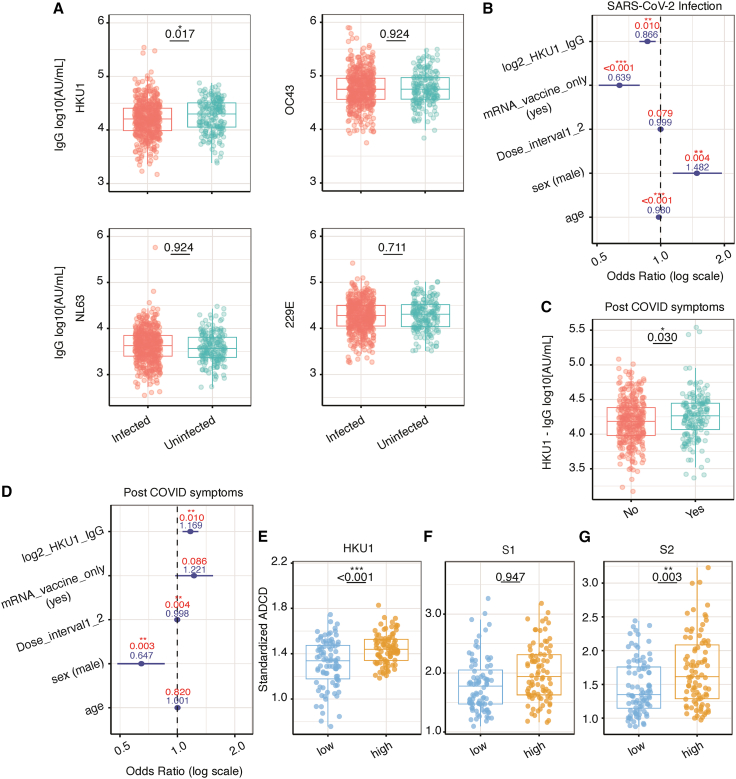
HKU1 antibody reactivity was protective against SARS-CoV-2 infection but increased the risk of post-COVID symptoms in vaccinated, infection-naive individuals (A) Comparison of HCoV spike IgG levels (HKU1, OC43, NL63, and 229E; log-transformed data) at baseline, between individuals infected and those who were not at the one-year follow-up. Comparisons were performed using a Wilcoxon rank-sum test (*n* = 700, infected = 509, uninfected = 191). (B) Effects of HCoV spike IgG levels, time interval between the first and second vaccine dose (days), use of mRNA vaccines only, sex, and age at baseline, on the risk of SARS-CoV-2 infection at the one-year follow-up. (C) Comparison of HCoV spike IgG levels at baseline, between individuals who developed post-COVID symptoms versus those who did not, by the one-year follow-up visit. Comparisons were performed using a Wilcoxon rank-sum test (*n* = 509, post-COVID symptoms = 165, no post-COVID symptoms = 335; missing data = 9). (D) Effects of HCoV spike IgG levels, time interval between the first and second dose (days), use of mRNA vaccines only, sex, and age at baseline, on the risk of post-COVID symptoms at the one-year follow-up. (B and D) Show adjusted odds ratios with 95% confidence intervals on a logarithmic scale (in blue) where the dashed horizontal line indicates no effect (OR = 1), with corresponding *p*-values (in red). (E–G) Antibody-dependent complement deposition (ADCD) between individuals with the highest (78^th^ centile, *n* = 88) and lowest (22^nd^ centile, *n* = 88) HKU1 spike (E), SARS-CoV-2 S1 (F) and S2 (G) IgG levels. Comparisons were performed using a Wilcoxon rank-sum test. For (A, C, E–G) data are shown as box (median, with lower/higher quartiles)-and-whiskers (upper whisker: Q3+1.5xIQR, lower whisker: Q1-1.5xIQR) plots, with all data points. For (B, D): Odds Ratios (OR) with 95% confidence intervals (blue line) and *p* value (in red), on a log scale. (∗*p* < 0.05, ∗∗*p* < 0.01, and ∗∗∗*p* < 0.001).

At the follow-up visit, 509 of 700 (72.7%) individuals had been newly infected during the one-year study period, as evidenced by a robust increase in nucleocapsid antibody levels (Figure S2A). We then determined how HKU1 spike IgG levels at baseline were associated with risk of infection at the one-year follow-up. Strikingly, HKU1 IgG levels at baseline were significantly higher in individuals who remained uninfected at the one-year follow-up visit, compared to those who were infected (*p* = 0.017), while no significant differences were detected for IgGs against other HCoVs (Figure 4A). The number of vaccine doses individuals had received at baseline was similar, supporting that the difference in HKU1 levels was not due to differences in vaccination rates between the two groups (Figure S2B).

To further explore this relationship, we conducted a multivariate binomial logistic analysis. Younger age and male sex were associated with a significant increase in infection risk during the follow-up period. The use of mRNA vaccines (as opposed to other COVID vaccines) was associated with a decreased infection risk. No significant associations were detected between dosing interval and infection risk. For HKU1, higher spike IgG levels significantly reduced SARS-CoV-2 infection risk after adjusting for age, sex, the type of vaccine received, and the time interval between doses (*p* = 0.003; Figure 4B). In contrast, other HCoVs (229E, OC43, and NL63) were not significantly associated with infection risk (Figures S2C–S2E). These findings suggest that pre-existing HKU1 IgG specifically protected against infection in this cohort.

Building on prior studies suggesting a potential detrimental role of HKU1 antibodies, particularly in the context of post-COVID symptoms and related neurological sequelae,^58^^,^^59^ we sought to investigate the impact of the HKU1 imprinting on post-COVID outcomes. Remarkably, individuals who reported post-COVID symptoms had significantly higher HKU1 spike IgG levels at baseline compared to those without symptoms (*p* = 0.030), whereas no statistically significant differences were observed for the other HCoVs (Figures 4C and S2F–S2H). The relationship between HKU1 spike IgG levels at baseline and the risk of post-COVID symptoms remained significant after adjusting for age, sex, dosing interval between the first and second dose, and the use of mRNA vaccines (*p* = 0.001; Figure 4D). These data suggest that pre-existing HKU1 IgGs increase susceptibility to post-COVID symptoms.

Given our earlier findings of enhanced Fc-mediated function among HKU1 cross-reactive antibodies, we hypothesized that these antibodies contribute to post-COVID through antibody-mediated complement deposition (ADCD).^60^ Infected individuals were stratified into two groups based on HKU1 spike IgG levels at the beginning of the one-year study period: 88 individuals with the highest HKU1 IgG levels and 88 individuals with the lowest levels (total *n* = 176) (Figure S2I). Sera from these groups were incubated with antigen-coated beads to capture antibodies targeting the HKU1 spike and SARS-CoV-2 S1 and S2 domains. We then measured ADCD on the resulting immune complexes.^61^ As expected, individuals with high HKU1 IgG levels showed significantly higher ADCD compared to individuals with low HKU1 IgG levels (Figure 4E). These findings linked high HKU1 levels with increased inflammatory Fc functions. Interestingly, no differences were observed for S1-specific antibody-mediated complement deposition between the two groups (Figure 4F), but the high HKU1 group showed greater S2-specific SARS-CoV-2 antibody-mediated complement deposition (Figure 4G). These results suggest that elevated HKU1 spike IgG is associated with enhanced Fc-mediated inflammatory functions of S2-specific antibodies. Overall, our findings suggest that high HKU1 IgG levels may confer protection against SARS-CoV-2 but paradoxically increase the risk of post-COVID symptoms through a heightened Fc-dependent complement-mediated inflammation.

## Discussion

In this study, we provide evidence that immune imprinting to β-HCoVs, particularly HKU1, shapes the immune response to SARS-CoV-2 mRNA vaccination. We show that a first dose of SARS-CoV-2 vaccination immediately recalls high avidity antibodies against β-HCoV spike (HKU1, OC43), that also show low avidity against the conserved S2 domain of SARS-CoV-2 spike. These findings are consistent with a recall response to β-HCoVs following infection or vaccination, as described by others.^35^^,^^37^^,^^41^^,^^48^ These antibodies also exhibited enhanced Fc effector functions that may facilitate early viral clearance but potentially promote pathological complement deposition, despite a low avidity against SARS-CoV-2 spike. Clinically, this translated into a reduced infection risk, but increased complement deposition and a greater likelihood of post-COVID symptoms in individuals with high HKU1-spike antibody levels.

This pattern is consistent with immune imprinting (“original antigenic sin”) that may constrain responses to antigenically novel variants.^40^^,^^46^^,^^62^^,^^63^ Consistently, ancestral SARS-CoV-2 vaccines boosted pre-existing HKU1 antibodies in two independent Vancouver cohorts. We extended these observations by demonstrating that vaccine-elicited recall responses predominantly involve low-avidity antibodies cross-reacting with β-HCoVs, and to a lesser extent, α-HCoVs. We also show that these cross-reactive B cells predominantly target the conserved S2 domain, which also shares greater sequence homology with β-HCoVs than with α-HCoVs.^27^^,^^28^^,^^29^^,^^30^ Our data are consistent with other studies showing an early increase in S2-specific antibodies after vaccination^62^^,^^63^^,^^64^ and support that the S2 domain is the primary target of HCoV-elicited pathological immune imprinting.^40^^,^^44^^,^^46^^,^^63^^,^^64^ The evidence of cross-reactive antibody responses specific to HKU1 in two independent cohorts from the same region, coupled to mechanistic studies, is a major strength of this study and supports reproducibility.

Although our studies show immune imprinting primarily associated with HKU1, similar imprinting may also arise with OC43.^65^ Greater specificity toward HKU1 may reflect a recent seasonal exposure to this specific HCoV in this population. Intriguingly, HKU1 predominated among individuals tested in the Vancouver metropolitan area in 2019 – the year preceding the COVID-19 pandemic – according to the British Columbia Center for Disease Control (Agatha Jassem, personal communication). This suggests HKU1-specific imprinting in the Vancouver cohorts reflects recent exposure to this HCoV. Alternatively, the predominance of HKU1-specific imprinting may also reflect intrinsic differences in viral evolutionary dynamics. Indeed, compared with OC43, HKU1 exhibits fewer circulating variants and thus lower genetic and antigenic diversification in the spike, consistent with a more conserved antigenic landscape.^66^ This may favor more abundant cross-reactive memory B-cell responses over time, thereby enhancing the detectability of HKU1-associated imprinting effects. In contrast, the greater genetic and antigenic diversification of the OC43 spike may diffuse immune memory across multiple variant-specific responses, reducing the apparent dominance of any single OC43-derived imprint.

The functions of S2 antibodies rely on their ability to interfere with the membrane-fusion required for viral entry and to engage Fc-mediated effector functions, such as antibody-dependent phagocytosis, cytotoxicity, or complement deposition.^67^^,^^68^^,^^69^^,^^70^^,^^71^^,^^72^ Here we provide further evidence that these antibodies can modulate vaccine outcomes through Fc-mediated effector functions. Importantly, while Fc-mediated functions are considered protective, an excess or a dysregulation of these functions has been implicated in inflammatory pathology, including in post-viral syndromes.^58^^,^^59^ Our study highlighted a dual role of cross-reactive antibodies in SARS-CoV-2 immunity: protecting against infection but paradoxically increasing the risk of developing post-COVID symptoms in infected individuals, a trend that may be counterproductive against the main objective of vaccines to keep recipients healthy.

The first vaccine dose induced an increase in HKU1 spike and S2, but not NL63 spike, antibody-mediated phagocytosis, suggesting that cross-reactive antibodies recognizing conserved motifs between SARS-CoV-2 and HKU1 may help promote early viral clearance. Consistent with this, our clinical data show that high levels of HKU1 antibodies reduced the risk of SARS-CoV-2 infection. However, paradoxically, among infected individuals, high levels of HKU1 spike antibody were associated with elevated antibody-mediated complement deposition, suggesting that Fc-potent cross-reactive antibodies may also drive excessive cellular toxicity during infection.^73^^,^^74^ These opposing effects may differ depending on the anatomical sites of responses and stage of infection. In the upper respiratory tract, protection likely arises from a combination of potent S1-RBD neutralizing antibodies that block viral entry and non-neutralizing S2-reactive antibodies that facilitate viral elimination through opsonization, phagocytosis, and other Fc-mediated effector mechanisms.^75^^,^^76^^,^^77^ However, if infection extends to the lower respiratory tract, where monocytes, macrophages, and NK cells expressing activating Fcγ receptors (FcγRIIa and FcγRIIIa) are frequent, we speculate that these Fc-potent IgG1 and IgG3 antibodies could form immune complexes that amplify complement activation, FcγR signaling, and pro-inflammatory cytokine release.^58^^,^^59^^,^^78^ This may explain why individuals with high HKU1 IgG are less likely to become infected but, once infected, exhibit greater risk of prolonged or inflammatory sequelae.

Whether cross-reactive antibodies arise from pre-existing memory B cells or through *de novo* B cell priming remains unclear. However, several lines of evidence support the recall of an established β-HCoV memory pool rather than the activation of naive or polyreactive B cells. Prior studies have demonstrated that cross-reactive antibodies targeting conserved S2 epitopes often exhibit extensive somatic hypermutation, indicative of antigen-experienced memory B cells rather than newly activated naive clones.^64^^,^^79^ Consistent with this, in our cohort, ADCP activity against S2 antigens and the HKU1 spike emerged as early as 14 days after the first vaccine dose, whereas S1-specific activity required revaccination. This observation aligns with a previous study reporting the detection of S2 cross-reactive antibodies by day 7 after the initial vaccine dose.^64^ Furthermore, another study showed that cross-reactive HCoV antibodies are largely IgM-depleted and predominantly IgGs, further supporting a recall of pre-existing cross-reactive antibody responses.^48^ Taken together, the kinetic pattern, especially considering the expected immunodominance of S1, strongly supports the activation of pre-existing β-coronavirus memory B cells rather than *de novo* priming of new polyreactive clones.

This study has public health implications. It highlights the need to determine whether boosters targeting more recent SARS-CoV-2 strains could reduce the risk of post-COVID symptoms in individuals who received only the primary ancestral vaccine series . While immune imprinting can offer broad protection, careful modulation of the immune response is needed to prevent unintended outcomes such as post-COVID syndromes as new variants emerge. This suggests that vaccination against more recent strains may be required, as ancestral-strain vaccines may be less effective at preventing post-COVID syndromes.

In conclusion, our study provides evidence that prior β-HCoV infections elicit immune imprinting toward SARS-CoV-2, shaping both antibody avidity and function. While this imprinting may protect against infection, it also appears to increase the risk of post-COVID symptoms through enhanced Fc-mediated inflammation in individuals vaccinated using an ancestral strain. These findings underscore the complex interplay between pre-existing and vaccine-induced immunity and emphasize the need for refined vaccine approaches that maximize protection while minimizing adverse outcomes. Further research is warranted to explore how immune imprinting shapes long-term immunity and how its benefits can be harnessed while mitigating potential drawbacks.

### Limitations of the study

First, the inference that HKU1 B cell reactivity reflects prior HKU1 exposure remains indirect and has not been directly investigated epidemiologically. Second, the associations observed between elevated HKU1 IgG levels and post-COVID symptoms do not establish causality, and further work is needed to clarify this relationship. One potential approach would be to perform detailed immunoprofiling of cellular responses to determine whether individuals with high HKU1 IgG titers display distinct inflammatory or regulatory signatures following infection, which could reveal downstream immune consequences of these antibody profiles. Complementary *in vitro* experiments could also assess how HKU1 spike-cross-reactive IgG influences cytokine production and immune cell activation, providing mechanistic insight into their functional effects. Additional studies examining Fc glycosylation, IgG subclass distribution, and mucosal IgA responses may help determine whether these antibody features modulate protective versus pathogenic outcomes. Finally, *in vivo* studies in mice expressing human Fc receptors could more directly test the impact of HKU1-cross-reactive antibodies on immune activation and tissue pathology, and whether they contribute to persistent inflammation or altered immune regulation after SARS-CoV-2 infection. Although our findings support the idea that S2-reactive memory B cells derive from pre-existing HKU1-specific immunity, this interpretation requires direct validation. Future studies integrating single-cell BCR sequencing, antigen mapping, and somatic hypermutation analyses will be essential to define the clonal and molecular basis of cross-reactivity between HKU1- and SARS-CoV-2–specific B cells.

## Resource availability

### Lead contact

Requests for further information and resources should be directed to and will be fulfilled by the lead contact, Pascal Lavoie (plavoie@bcchr.ca).

### Materials availability

This study did not generate new unique reagents.

### Data and code availability

• Raw data have been deposited at Mendeley Data and are publicly available as of the date of publication at Mendeley Data: https://doi.org/10.17632/rhd4mbn2cg.1.
• This article does not report original code.
• Any additional information required to reanalyze the data reported in this article is available from the lead contact upon request.

## Acknowledgments

We thank all the study participants who contributed blood samples for these studies. AM was supported by a Mining for Miracles postdoctoral scholarship from the 10.13039/100011775British Columbia Children’s Hospital Foundation (BCCF). PML and LCM received salary support from the 10.13039/100000994BCCF through the Investigator Grant Award Program. This work was supported by the Government of Canada’s COVID-19 Immunity Task Force (to PML; award # AWD-016994) and by a Project Grant from the 10.13039/501100000024Canadian Institutes of Health Research (to PML: PJT-166103).

## Author contributions

A.M. and P.M.L. designed the study. A.M., C.M., A.W.W., and L.C.M. contributed to the study cohorts’ recruitment and sample collection. BAR and FR optimized the antibody avidity assays. A.M. performed the immunology experiments. S.L. and J.S. contributed to the ADCD experiments. X.D. and A.G. contributed to data analyses. A.W.W. contributed to the analysis of the second cohort. A.M. and P.M.L. wrote the article draft. All other authors revised it critically for important intellectual content. Funding was obtained by P.M.L. and L.C.M. All authors approved the final version to be published and are in agreement to be accountable for all aspects of the work in ensuring that questions related to the accuracy or integrity of any part of the work are appropriately investigated and resolved. A.M. and P.M.L. had full access to all the data.

## Declaration of interests

The authors declare no competing interests.

## Declaration of generative AI and AI-assisted technologies in the writing process

During the preparation of this work, the author(s) used ChatGPT to identify grammatical errors in the text. Once identified, the errors were manually corrected, if applicable, by the author. After using this tool/service, the author(s) reviewed and edited the content as needed and take(s) full responsibility for the content of the published article.

## STAR★Methods

### Key resources table

**Table:** 

REAGENT or RESOURCE	SOURCE	IDENTIFIER
**Antibodies**		

Anti-CD19-BV650	BioLegend	Cat# 302238; RRID:AB_2562097
Anti-CD3-PerCP-Cy5.5	BioLegend	Cat# 317336; RRID: AB_2561628
Anti-CD14-PerCP-Cy5.5	BioLegend	Cat# 325622; RRID: AB_893250
Anti-CD56-PerCP-Cy5.5	BioLegend	Cat# 318322; RRID: AB_893389
Anti-IgG-BUV805	BD Biosciences	Cat# 742041; RRID: AB_2871333
Anti-IgM-AF700	BioLegend	Cat# 314544; RRID: AB_2800832
anti-IgA-FITC	Miltenyi Biotec	Cat# 130-113-475; RRID: AB_2726166
Anti-IgD-BUV395	BD Biosciences	Cat# 566243 RRID: AB_2739624
Anti-CD38-BUV661	BD Biosciences	Cat# 612970; RRID: AB_2916888
Anti-CD27-PE	BioLegend	Cat# 302808; RRID: AB_314300
Anti-CD24-APC-Cy7	BioLegend	Cat# 311132; RRID: AB_2566347
Goat anti–guinea pig C3 fluorescein antibody	MP Biomedicals	Cat# 0855385; RRID: AB_2334913

**Chemicals, peptides, and recombinant proteins**		

Fixable viability dye eFluor506	Thermo Fisher	Cat# 65-0866-14
Lymphoprep	STEMCELL Technologies	Cat# 07861
Duman serum albumin	Gemini Bio	Cat# 800-120
Dimethyl sulfoxide (DMSO)	MilliporeSigma	Cat#D8418
Ammonium thiocyanate	MilliporeSigma	Cat# A1479
EDTA 15 mM	Invitrogen	Cat# AM9260G
Biotinylated SARS-CoV-2 S1	Sino Biological	Cat# 40591-V08H-B
Biotinylated SARS-CoV-2 S1	CROBiosystems	Cat# S1N-C82E8
Biotinylated SARS-CoV-2 S2	Sino Biological	Cat# S2N-C52E8
Biotinylated HKU1 spike	Sino Biological	Cat# 40606-V08B-B
Streptavidin–APC	BD Biosciences	Cat# 563259
Streptavidin–BV421	BD Biosciences	Cat# 405237
Benzonase® Nuclease	MilliporeSigma	Cat# 70746
Guinea pig complement	Cedarlane	Cat# CL4051
Biotinylated HKU1 spike	Sino Biological	Cat# 40606-V08B-B
Biotinylated NL63 spike	Sino Biological	Cat# 40604-V08B-B
Paraformaldehyde 4%	Thermo Scientific	Cat# J61899.AK

**Critical commercial assays**		

IgG multiplex assay	Meso Scale Discovery	Cat# K15369U

**Deposited data**		

All raw data	This paper	10.17632/rhd4mbn2cg.1

**Software and algorithms**		

FlowJo v10	BD Biosciences	–
Discovery Workbench v4.0	Meso Scale Discovery	–

**Other**		

BD Vacutainer tube	Becton Dickinson	Cat# 367989
BD Vacutainer EDTA tubes	VWR	Cat# CABD367861L
SepMate™ PBMC Isolation Tubes	STEMCELL Technologies	Cat# 85460
NeutrAvidin beads (red)	Thermo Fisher	Cat# F8775
NeutrAvidin beads (yellow-green)	Thermo Fisher	Cat# F8776

### Experimental model and participant details

#### Study participants

The first cohort included uninfected healthcare workers with no history of SARS-CoV-2 infection and a negative spike serology (by total IgA, IgG, and IgM against recombinant spike [S1] using the VITROS 5600 analyzer; Ortho-Clinical Diagnostics) recontacted (*N* = 96 approached for consent) from a previous study,^47^ enrolled between February and June 2021 (*N* = 48 participants enrolled, 35 females), who received their primary SARS-CoV-2 vaccine series against the ancestral strain between January and August 2021. The standard interval between the first and second vaccine doses in Canada at the time was 3 months. Participants provided a baseline blood sample within 14 days before receiving the first SARS-CoV-2 vaccine dose, and blood samples at 14 days and 28 days after the first and second vaccine doses.

The second cohort included a representative subset of 700 school workers (581 females) enrolled from three school districts in the Greater Vancouver area, BC, Canada, with the baseline blood sample collected between January and April 2022, and the second blood sample collected at the one-year follow-up between January and April 2023.^56^^,^^80^ Only fully vaccinated participants who showed no evidence of prior SARS-CoV-2 infection, based on negative nucleocapsid serology on the baseline sample (using the Elecsys Anti-SARS-CoV-2 anti-nucleocapsid assay; Roche, USA, on a Cobas e601 analyser) were included in the second cohort analysis. The basic characteristics of individuals enrolled in the two cohorts are presented in Table S1. Gender, ancestry, race, ethnicity and socioeconomic status were not captured.

Written informed consent was obtained from all participants. All studies were conducted in accordance with relevant Canadian regulations and guidelines, including the Tri-Council Policy Statement: Ethical Conduct for Research Involving Humans (TCPS 2), and were approved by the University of British Columbia (UBC) C&W Research Ethics Board (H20-01205; H18-01724).

### Method details

#### Sample processing

Blood for serum preparation was collected at BC Children’s Hospital Research Institute in a BD Vacutainer serum tube (Becton Dickinson, Franklin Lakes, NJ, USA; cat# 367989). Samples were incubated for 30 min at room temperature and then centrifuged at 1400g for 10 min prior to aliquoting sera, for storage at −80 °C. Blood for Peripheral Blood Mononuclear Cells (PBMCs) was collected at the same time, in a 10 mL BD Vacutainer EDTA tube (VWR, Mississauga, ON, Canada; cat# CABD367861L). For PBMC isolation by gradient centrifugation, the blood was diluted 1:1 with phosphate-buffered saline (PBS) and layered on top of 15 mL Lymphoprep (STEMCELL Technologies, Vancouver, BC, Canada; cat# 07861) in SepMate PBMC Isolation Tubes (STEMCELL Technologies, Vancouver, BC, Canada; cat# 85460). After centrifugation for 10 min at 1200g with brake on, the mononuclear cell layer was harvested and washed twice with PBS before cryogenic storage in liquid nitrogen in the presence of human serum albumin (Gemini Bio, West Sacramento, CA, USA) and 10% dimethyl sulfoxide (DMSO).

#### SARS-CoV-2 infection status

Prior SARS-CoV-2 infections in both cohorts were ascertained using the history of SARS-CoV-2 viral testing, and annual spike serologies done as part of the parent longitudinal,^56^^,^^57^^,^^80^ measured by total IgA, IgG, and IgM against recombinant spike (S1) on the VITROS 5600 analyzer (Ortho-Clinical Diagnostics) and the Elecsys Anti-SARS-CoV-2 nucleocapsid assay (Roche, USA) on a Cobas e601 analyzer.

#### Multiplex antibody assay

A multiplex assay (Meso Scale Discovery, Rockville, MD, USA; cat# K15369U) was used to measure antibody profiles. After blocking with 5% bovine serum albumin (BSA, Millipore Sigma-Aldrich, ON Canada), sera were diluted 1:10,000 and incubated with shaking for 2 h. Sulfo-tag–labeled anti-IgG detection antibodies were added, and signals were read using a Sector S 600 instrument (Meso Scale Discovery MD USA). Results were expressed as dilution-corrected values in AU/mL, calibrated to an International Standard Serum (ISS) by Meso Scale Diagnostics.

#### Fractional avidity assay

Avidity was measured using ammonium thiocyanate (NH_4_SCN, ≥98.9%, MilliporeSigma, MA, USA; cat# A1479) in serial dilutions. After serum binding on Meso Scale Discovery plates (Meso Scale Discovery MD USA; cat# K15369U), chaotropic solutions were applied at 0, 0.5, 0.75 and 2.0 M. Detection used Sulfo-tag–labeled anti-IgG and the MSD Sector 600 instrument. Data were processed with Discovery Workbench v4.0 (Meso Scale Discovery). Avidity fractions (AFA) were calculated as: AFA_n_ = Y_n_ − Y_n+1_.

#### Flow cytometry analysis of B cells

Biotinylated SARS-CoV-2 S1 and S2 proteins were purchased from either Sino Biological, Beijing, China (cat# 40591-V08H-B), or ACROBiosystems, Newark, DE, USA (cat# S1N-C82E8 and S2N-C52E8, respectively). Each protein was double conjugated to an equimolar amount of streptavidin–APC (BD Biosciences, San Jose, CA, USA; cat# 563259) and streptavidin–BV421 (BD Biosciences, San Jose, CA, USA; cat# 405237). For HKU1-specific B cells, biotinylated HKU1 spike proteins were purchased from Sino Biological, Beijing, China (cat# 40606-V08B-B). Frozen PBMCs were washed to remove DMSO and rested at 37 °C for 1 h, followed by incubation with DNase (Benzonase Nuclease, MilliporeSigma, Burlington, MA, USA; cat# 70746) for 15 min. Cells were then washed and incubated in PBS with the following anti-human antibodies (all at 1:200 dilution): anti-CD19-BV650 (BioLegend, San Diego, CA, USA; cat# 302238), anti-CD3-PerCP-Cy5.5 (BioLegend, cat# 317336), anti-CD14-PerCP-Cy5.5 (BioLegend, cat# 325622), anti-CD56-PerCP-Cy5.5 (BioLegend, cat# 318322), anti-IgG-BUV805 (BD Biosciences, cat# 742041), anti-IgM-AF700 (BioLegend, cat# 314544), anti-IgA-FITC (Miltenyi Biotec, Bergisch Gladbach, Germany; cat# 130-113-475), anti-IgD-BUV395 (BD Biosciences, cat# 566243), anti-CD38-BUV661 (BD Biosciences, cat# 612970), anti-CD27-PE (BioLegend, cat# 302808), anti-CD24-APC-Cy7 (BioLegend, cat# 311132), as well as APC- and BV421-conjugated streptavidin-tagged S1 or S2 proteins and fixable viability dye eFluor506 (eBioscience, Thermo Fisher Scientific, Waltham, MA, USA; cat# 65-0866-14). After 20 min of incubation in the dark under agitation (700 rpm) at room temperature, cells were washed twice, fixed in 4% paraformaldehyde (PFA) for 10 min, washed again, and acquired on a BD FACSymphony flow cytometer. Data were analyzed using FlowJo software (BD, Ashland, OR USA). Our approach of gating strategy of B cells using CD24 allows to analyze the broader pool of antigen-experienced B cells without excluding populations that may upregulate CD24, such as transitional and certain activated or resting memory B cells, depending on their activation state and tissue origin.^81^^,^^82^

#### Antibody dependent cellular phagocytosis (ADCP)

Ten μL of yellow-green fluorescent NeutrAvidin-labeled microspheres (Thermo Fisher Scientific, Waltham, MA, USA; cat# F8776) were washed with PBS containing 0.1% BSA, resuspended in 10 μL of biotinylated SARS-CoV-2 S1, S2 (ACROBiosystems, cat# S1N-C82E8 and S2N-C52E8), HKU1 (Sino Biological, cat# 40606-V08B-B), and NL63 (Sino Biological, cat# 40604-V08B-B) spike proteins (0.1 μg/μL), and incubated at 36 °C for 2 h. Microspheres were washed twice, resuspended in 1 mL PBS, and aliquoted (10 μL) into a 96-well plate. Equal volumes (10 μL) of 1:200 diluted heat-inactivated sera were added, followed by 2 h of incubation at 36 °C and two PBS washes. Finally, 180 μL of 5×10^4^ THP-1 cells in serum-free medium (STEMCELL Technologies, cat# 10981) were added to each well and incubated at 30 °C for 12 h. THP-1 cells were washed, stained with fixable viability dye eFluor780 (eBioscience, Thermo Fisher Scientific; cat# 65-0865-14) for 10 min at room temperature in the dark, washed with PBS, fixed in 4% PFA for 10 min, and washed again prior to acquisition on a CytoFLEX flow cytometer (Beckman Coulter, Brea, CA, USA). Data were analyzed using FlowJo software version 10 and expressed as ADCP score = % FITC^+^ cells × geometric MFI of FITC^+^ cells.

#### Antibody dependent complement deposition (ADCD)

ADCD was performed as previously described.^58^ Briefly, 10 μL of red fluorescent NeutrAvidin-labeled microspheres (Thermo Fisher Scientific, cat# F8775) were washed with PBS–0.1% BSA, resuspended in 10 μL of biotinylated SARS-CoV-2 S1, S2 (ACROBiosystems), HKU1 (Sino Biological; cat# 40606-V08B-B) (0.1 μg/μL), and incubated at 37 °C for 2 h. Microspheres were washed, resuspended in 1 mL PBS, and aliquoted (10 μL) into a 96-well plate. Equal volumes of diluted sera (1:200) were added and incubated at 36 °C for 2 h, then washed twice with PBS. Lyophilized guinea pig complement (Cedarlane, Burlington, ON, Canada; cat# CL4051) was reconstituted in distilled water, heat-inactivated at 56 °C for 30 min, and centrifuged at 16,000g for 5 min at 4 °C. Complement was diluted in RPMI with 10% FBS (Gibco, Thermo Fisher Scientific) at 1:50, and 200 μL was added to each well. After 15 min of incubation at 37 °C, beads were pelleted and washed twice with 15 mM EDTA in PBS (Invitrogen, Thermo Fisher Scientific; cat# AM9260G). Beads were incubated with 50 μL of fluorescein-conjugated goat anti-guinea pig C3 antibody (MP Biomedicals, Irvine, CA, USA; cat# 0855385) for 15 min at room temperature, washed, resuspended in PBS, fixed with 4% PFA (Santa Cruz Biotechnology, Dallas, TX, USA; cat# sc-281692), and analyzed by flow cytometry. Data were processed using FlowJo software.

#### Assessment of post-COVID symptoms

Post-COVID symptoms were assessed in the second school worker cohort, using a standardized questionnaire adapted from Vancouver Coastal Health’s post-COVID-19 recovery clinic, as described.^57^ Participants who self-reported a prior SARS-CoV-2 infection were asked: *“Have you experienced long-lasting symptoms of more than three months that you did not have prior to having COVID-19?”* Those responding “yes” were presented with a predefined list of symptoms and asked to indicate whether each symptom was current or had occurred previously. The symptom list included: persistent fatigue, decreased energy to exercise, shortness of breath, chest pain, abdominal pain, palpitations, trouble sleeping, headache, weakness, loss of taste or smell, hoarse voice or voice change, rashes, discoloration of fingers or toes, dizziness, numbness or paresthesia, difficulty concentrating or forgetfulness (“brain fog”), and an “other” category for additional symptoms. Participants selecting any symptom were classified as having chronic or post-COVID symptoms. Additional questions assessed the duration and severity of the of symptoms (see: https://bmjopen.bmj.com/content/bmjopen/15/7/e095685/DC1/embed/inline-supplementary-material-1.pdf?download=true for the original questionnaire).

### Quantification and statistical analysis

Differences between groups or across timepoints were evaluated using the Mann–Whitney U test for unpaired comparisons and the Wilcoxon signed-rank test for paired comparisons. Two-sided tests were used unless otherwise specified. When repeated-measures data contained missing observations across timepoints, mixed-effects models were used to appropriately account for missing data across timepoints. Models were fitted using the lmer() function with participant ID included as a random intercept, followed by estimated marginal means and pairwise contrasts generated using the emmeans package.

Spearman correlations were used to assess relationships among ADCP across the vaccination timeline, for each study timepoint at baseline, 14 and 28 days after dose 1, and 14 and 28 days after dose 2. Pairwise correlation coefficients were calculated using complete observations. Statistical significance was assessed using pairwise correlation tests applying the Benjamini–Hochberg false discovery rate (FDR)-adjusted *p*-values (5%). Heatmaps were generated using the corrplot package, employing a diverging color scale and removing the diagonal for clarity. Significant correlations were annotated using asterisks (∗*p* < 0.05, ∗∗*p* < 0.01, ∗∗∗*p* < 0.001).

Multivariable logistic regression models were fitted using infection status and post-COVID symptoms as the binary outcome to identify baseline immune features associated with subsequent SARS-CoV-2 infection. Predictors in the model included: log2-transformed IgG titers, dose interval between the first two vaccine doses, mRNA vaccination status, age and sex. Models were fitted using the glm() function with a binomial in “R”. Vaccination status at the end of study was used as a probability weight in all logistic regression models to correct for sampling imbalance driven by the possible difference between groups in terms of vaccine dose numbers. Model results were visualized using forest plots on a log scale, displaying odds ratios, confidence intervals, and significance levels. All statistical analyses were performed in R (version 4). Statistical significance was defined as *p* < 0.05.
