# Supplementary material for: HKU1 immune imprinting is associated with post-COVID symptoms after SARS-CoV-2 infection
Source: iScience. 2026 Mar 2;29(4):115175. doi: 10.1016/j.isci.2026.115175 (PMC12999348; doi:10.1016/j.isci.2026.115175)
Supplement: Document S1. Figures S1 and S2 and Table S1 [file mmc1.pdf]

iScience, Volume *29*

Supplemental information

HKU1 immune imprinting is associated with post-COVID symptoms after SARS-CoV-2 infection

Abdelilah Majdoubi, Christina Michalski, Allison W. Watts, Xiaoqing Dang, S. Amirhossein Golzan, Bahaa Abu-Raya, Sirui Li, Jacob Shew, Frederic Reicherz, Louise C. Mâsse, and Pascal M. Lavoie

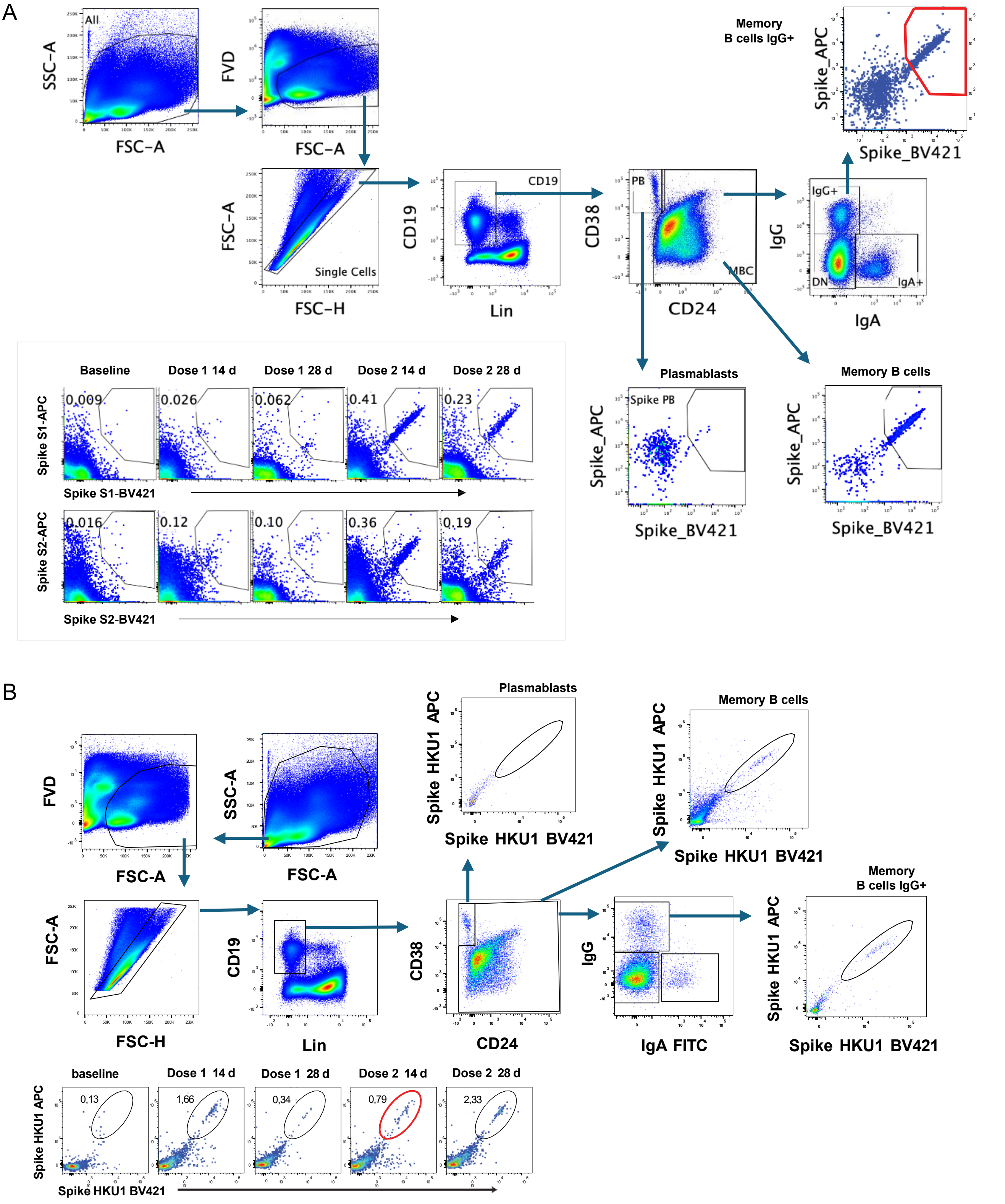

Figure S1: Flow cytometry gating strategy for SARS-CoV-2 S1, S2 and HKU1-specific B cell subsets. (**A**) Gating strategy for S1 and S2-specific and (**B**) HKU1 B cells with corresponding representative dot plots for double spike staining across vaccine timepoints (baseline, 14 and 28 days after 1st dose, and 14 and 28 days after 2nd dose). Peripheral blood mononuclear cells (PBMCs) were first gated based on forward scatter area (FSC-A) and side scatter area (SSC-A) to select lymphocytes. Dead and doublets cells were excluded using a fluorescent viability dye (FVD) and the FSC-A/FSC-H gate, respectively. Singlet B cells were identified as $CD19^{+}$ and lineage-negative (Lin^{-}; NK, CD14, CD3) cells. Plasmablasts were defined as $CD38^{high}$ $CD24^{-}$ cells, and the remaining cells were classified as B cells, which included memory B cells (MBCs) and IgG+ MBCs.

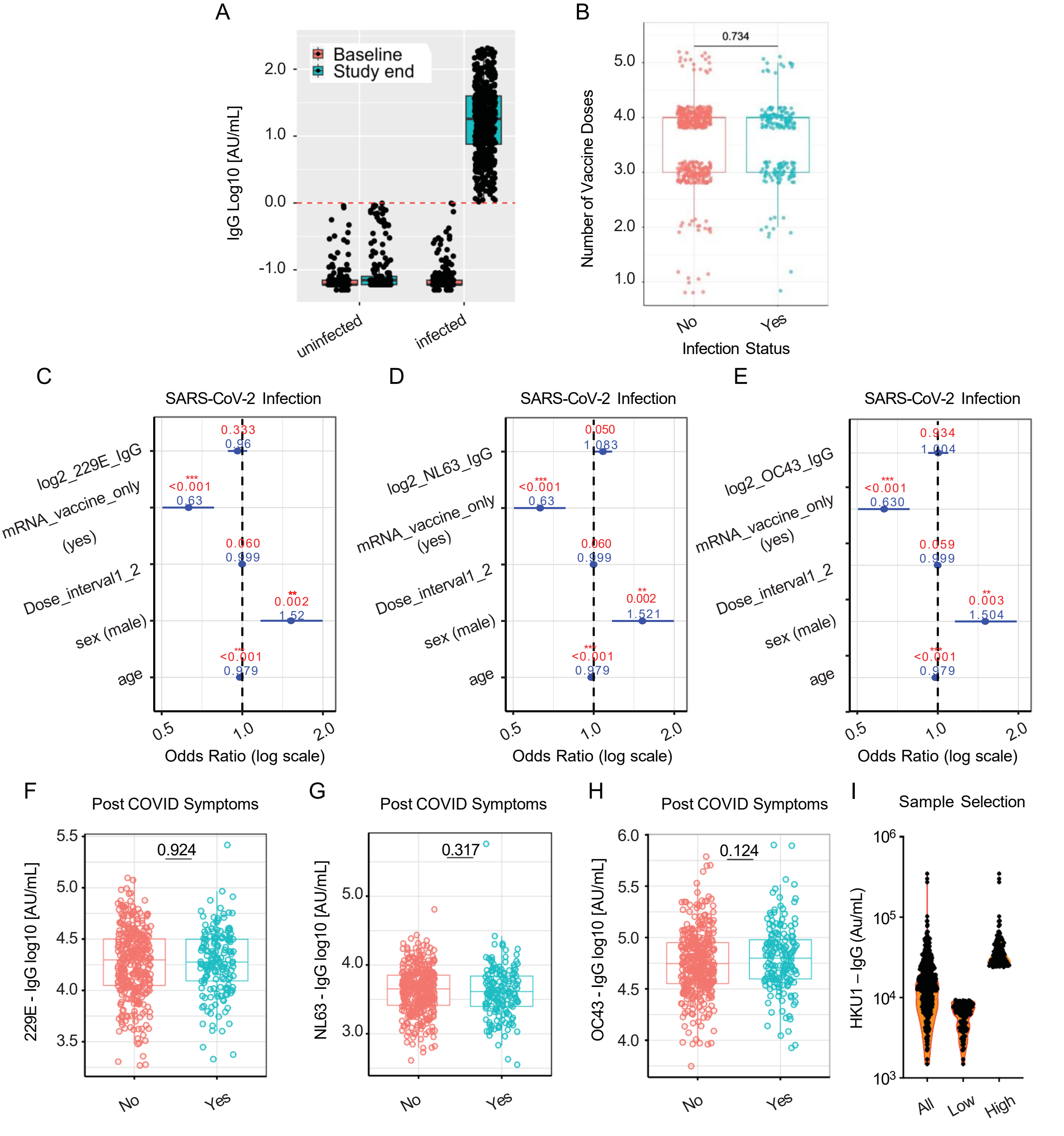

Figure S2. HKU1 associations with SARS-CoV-2 infection and post-COVID symptoms. (**A**) Changes in SARS-CoV-2 nucleocapsid-specific IgG serology measured at baseline and at the end of the one-year study period (follow-up) among school workers (cohort 2), comparing individuals who remained uninfected with those who became infected by the end of follow-up. (**B**) Number of vaccine doses received at the beginning of the study period. Differences in vaccine dose number at baseline, between participants who were infected versus uninfected at follow-up were assessed using a two-sided Mann–Whitney U test. Odds ratios for post-COVID symptoms associated with baseline IgG levels against the spike proteins of (**C**) 229E, (**D**) NL63, and (**E**) OC43, adjusted for age, sex, mRNA vaccine use, and the interval between vaccine doses 1 and 2. Analyses were weighted according to the number of vaccine doses received. Odds ratios (OR) adjusted for covariates are shown with 95% confidence intervals on a logarithmic scale; the dashed horizontal line indicates OR = 1 (no association), with corresponding p values. Comparisons of IgG levels against (**F**) 229E, (**G**) NL63, and (**H**) OC43 spike proteins between infected individuals who developed post-COVID symptoms and those who did not were performed using a two-sided Mann–Whitney U test. (**I**) Sample selection based on HKU1-specific IgG levels: 88 samples were selected from individuals with low (22nd percentile) and high (78th percentile) HKU1 IgG levels.($^*P < 0.05$, $^{**}P < 0.01$, $^{***}P < 0.001$). For (A, B, F-I) all data points are shown, with box (median, with lower/higher quartiles)-and-whiskers (upper whisker: Q3+1.5xIQR, lower whisker: Q1-1.5xIQR) plots.

Table S1. Basic characteristics of individuals in each cohort.

	Cohort 1 (healthcare workers) N = 48	Cohort 2 (school workers) N = 700	
		Infected N = 509	Uninfected N = 191
Age, mean ± SD	40.0 ±10.87	48.2 ± 10.0	50.7 ± 9.0
Sex, n (%) female	35 (72.9)	417 (81.9)	164 (85.9)
Interval dose 1 to dose 2 (days), median (IQR)	98 (95 – 104)	68 (60 – 121)[õ]	71 (61 – 182)
% mRNA vaccine only	100%	308 (60.9)	131 (68.6)[£]
Post-COVID symptoms	NA	165 (33.0)[*]	NA

[õ]Data were missing for 17 individuals

[*]Data were missing for 9 individuals

[£]Data were missing for 3 individuals
